# Carbon Nanotube Reinforced High Density Polyethylene Materials for Offshore Sheathing Applications

**DOI:** 10.3390/molecules25132960

**Published:** 2020-06-27

**Authors:** Chinyere Okolo, Rafaila Rafique, Sadia Sagar Iqbal, Mohd Shahneel Saharudin, Fawad Inam

**Affiliations:** 1 Department of Mechanical and Construction Engineering, Northumbria University, Newcastle upon Tyne NE1 8ST, UK; chinyere.okolo@northumbria.ac.uk; 2 H.E.J. Research Institute of Chemistry, International Centre for Chemical and Biological, Sciences, University of Karachi, Karachi 75270, Pakistan; rafailasiddique@hotmail.com; 3 Department of Physics, University of Lahore, Lahore 54590, Pakistan; sadia.pet.ceet@pu.edu.pk; 4 Malaysia Italy Design Institute (UniKL MIDI), Universiti Kuala Lumpur, Kuala Lumpur 56100, Malaysia; mshahneel@unikl.edu.my; 5 Department of Engineering and Computing, University of East London, London E16 2RD, UK

**Keywords:** polyethylene, carbon nanotubes, nanocomposites, offshore engineering, sheathing, applications

## Abstract

Multiwall carbon nanotube (CNT)-filled high density polyethylene (HDPE) nanocomposites were prepared by extrusion and considered for their suitability in the offshore sheathing applications. Transmission electron microscopy was conducted to analyse dispersion after bulk extrusion. Monolithic and nanocomposite samples were subjected to accelerated weathering and photodegradation (carbonyl and vinyl indices) characterisations, which consisted of heat, moisture (seawater) and UV light, intended to imitate the offshore conditions. The effects of accelerated weathering on mechanical properties (tensile strength and elastic modulus) of the nanocomposites were analysed. CNT addition in HDPE produced environmentally resilient nanocomposites with improved mechanical properties. The energy utilised to extrude nanocomposites was also less than the energy used to extrude monolithic HDPE samples. The results support the mass substitution of CNT-filled HDPE nanocomposites in high-end offshore applications.

## 1. Introduction

**Figure 1:**
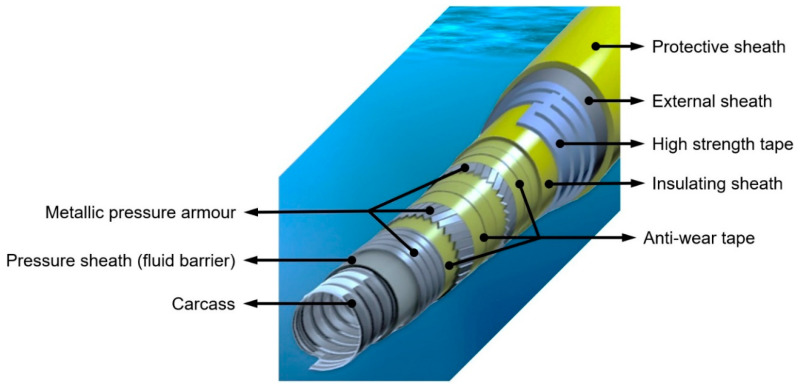
Schematic of a typical flexible umbilical cable for offshore engineering.

With oil and gas exploration moving towards deeper oceans with harsher thermo-mechanical environments, it has become ever so critical to find economical materials and umbilical systems providing appropriate tensile stiffness to withstand large loads induced by self-weight, sea current and the motion of the surface vessel. Umbilical cable (Figure 1) is an offshore product, utilised for the subsea oil and gas exploitation and related projects. The cable contains a complex and telescopic hierarchy of tubes/hoses, optical fibre cables, electrical cables, inner sheaths and fillers which are assembled into an inner core [1]. The main aim of the cable is to provide a control and communication channel between the surface vessel and the subsea installations and equipment. The macro-composite structure contains multiple components and various types of engineering materials. Ferrous materials, such as steel armour wires, are used to provide the tension capability and to achieve the necessary internal stability against the high hydrostatic pressure [1].

Polyethylene (PE), consisting of ethylene monomers ((C_2_H_4_)_n_), is one of the simplest structured and commonly used plastics for a number of engineering applications, including for sheathing (inner and external, Figure 1) purposes in umbilical cables [1,2]. In particular, one of the commonly used materials for these advanced applications is high density polyethylene (HDPE), owing to its high strength-to-density ratio.

Incorporation of carbon nanotubes (CNTs) in high-density polyethylene (HDPE) [3,4,5,6,7,8,9,10] and other polymers [11,12,13] has been widely explored and appreciated. CNTs were discovered in 1991 [14] as one of the by-products of fullerene synthesis. Remarkable progress has been made since then, including the discovery of several types of nanotubes (single-wall and multiwall) and over last two decades. Significant research has been conducted for their synthesis and purification, and the elucidation of their fundamental physical properties; and realistic steps are being taken towards their substitution into key engineering applications [15,16,17].

From the analysis of the literature over nearly the past three decades, there are not many related research efforts looking into this practical application. Studies by Tang et al. [6] prepared composite films with 0%, 1%, 3% and 5% of CNT content (by weight). They reported an increase in elastic modulus (up to 8%), peak load (up to 13%) and work to failure (up to 5%) for the composite films with increasing CNT content. Achaby and Qaiss [7] prepared HDPE/CNT nanocomposites using the melt mixing technique and compared their thermo-mechanical properties with HDPE/graphene nanocomposites. They reported superior elastic modulus (up to 87% in comparison to neat HDPE), tensile strength (up to 77% in comparison to neat HDPE) and thermal stability for HDPE/graphene nanocomposites owing to the higher specific surface area, larger aspect ratio and nanoscale 2D flat surface of graphene. However, there was no analysis or discussion on the fibrous nature of CNTs, which, when aligned in the direction of extrusion, could enhance the tensile properties required for the umbilical cable application (Figure 1). Fouad et al. [9] prepared a series of HDPE/CNT nanocomposites using the melt blending technique and analysed the morphological, thermal, rheological, viscoelastic, mechanical and fracture toughness properties of the nano-composites. The group reported 4 wt% as the most optimal loading percentage for improved storage modulus, Young’s modulus (up to 46%) and yield strength (up to 10%) of the nanocomposites with increasing CNT content. However, there was no analysis on the degradation of the nanocomposites. In another study, Du et al. [18] processed HDPE/CNT and HDPE/graphene nanocomposites by alcohol-assisted dispersion and hot-pressing and compared dispersion and electrical properties. Due to the formation of crimps, the rolling and the aggregation of graphene in the HDPE matrix, the two-dimensional graphene was not as effective as multi-wall CNTs (MWCNTs) at forming conductive/electrical networks and resulted in poor dispersion as well. Similarly, Ferreira et al. [19] and Johnson et al. [20] reported improved hardness and wear resistance respectively when CNTs were dispersed in HDPE. In 2007, Kanagaraj et al. [21] reported improved toughness (up to 33%), modulus (up to 22%), tensile strength (up to 4%) and strain to failure (up to 24%) by adding small amounts on CNTs; i.e., less than 0.45 vol%. Che et al. [22] prepared ternary composites by adding CNTs and expanded graphite in HDPE. They reported significant rises in electrical and thermal conductivities, tensile strength (up to 84%) and Young’ modulus (up to 25%) without commenting on any weathering effects. Similarly, Mokashi et al. [23] and Okolo et al. [16] also found improvements in the tensile strength and elastic stiffness of CNT-filled polyethylene nanocomposites. Kodije et al. [24] studied the morphology, crystallisation behaviour and thermal stability of HDPE/CNT nanocomposites and reported improved thermal stability for the nanocomposites. However, it was a laboratory study prepared by solution blending technique and there was no analysis of the mechanical properties. Mohsin et al. [25] added mixed fillers, such as montmorillonite and maleic-anhydride-grafted high density polyethylene to HDPE/CNT nanocomposites and reported enhanced mechanical properties without affecting the basic thermal properties (crystallisation and melting temperatures) of the nanocomposites. From the processing perspective, Zou et al. [8] concluded that HDPE/CNT nanocomposites fabricated at higher screw speed give uniform dispersion of CNT in HDPE. Other studies by Xiang et al. [10] analysed the influences of processing route on the mechanical properties of the nanocomposites; the authors used a combination of compression moulding and blown film extrusion techniques.

However, CNT-based materials are very much lost in the research domain, where we are finding a number of papers without any evidence-supported commentary on industrial application or real impact. This work is the first study to explore the utilisation of CNT-based HDPE nanocomposites for offshore umbilical cable applications. None of the previous studies analysed holistically within the context of umbilical cable (Figure 1) applications used for offshore oil and gas exploration. This research looked into degradability in offshore conditions and the relevant mechanical properties desirable for umbilical hoses for deeper waters. The current research also examined the manufacturing economics, providing a comprehensive materials substitution analysis for the presently and commonly used HDPE materials by the incorporation of CNTs.

## 2. Results and Discussion

**Figure 2:**
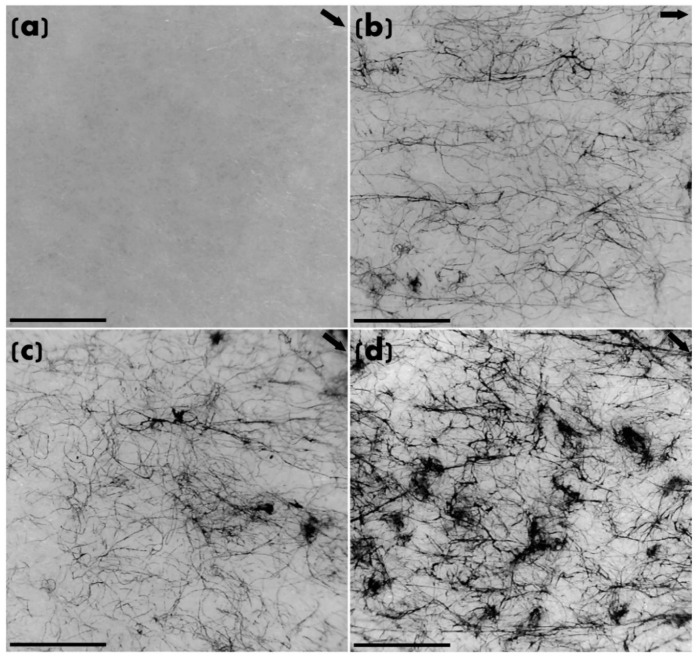
Representative TEM images for: (**a**) monolithic HDPE; and nanocomposites containing: (**b**) 2 wt% CNTs; (**c**) 4 wt% CNTs; and (**d**) 6 wt% CNTs. Direction of extrusion is represented by the arrow on the top right. Scale bar represents 500 nm.

TEM analysis was carried out to investigate the dispersion, distribution and orientation of CNTs in HDPE nanocomposites prepared using different filler content and their correlation in relation to the monolithic HDPE, as shown in Figure 2. The relatively low TEM magnification was strategically used to analyse a larger area for analysis of homogenisation at higher resolution to achieve an accurate representation. Whilst at high magnification, one can clearly resolve individual CNTs, the approach does not show the representative factual picture of overall dispersion, for which it is appropriate to select a magnification where fibre-like morphology could be observed and overall homogenisation can be assessed (Figure 2). There are varying regions of CNT aggregates and individual and sparse CNTs (Figure 2b–d). The conventional melt mixing (extrusion) is not effective at deagglomerating and homogenously dispersing CNTs at such a high loading of 6 wt% in HDPE matrix (Figure 2d). Some aggregates can also be seen in the representative image for 4 wt% CNT filled HDPE nanocomposite (Figure 2c). However, it can be concluded that the CNT dispersion/exfoliation is much better in the nanocomposites with low CNT loadings (Figure 2b,c) where the fibre-like morphology of individual CNTs can be seen as well. Direction of extrusion is represented by the arrow on the top right of all the images (Figure 2). In comparison to monolithic HDPE (Figure 2a), CNTs (and their aggregates) seem to be aligned in the direction of extrusion, which was also observed in other reports [26,27,28] as well.

**Figure 3:**
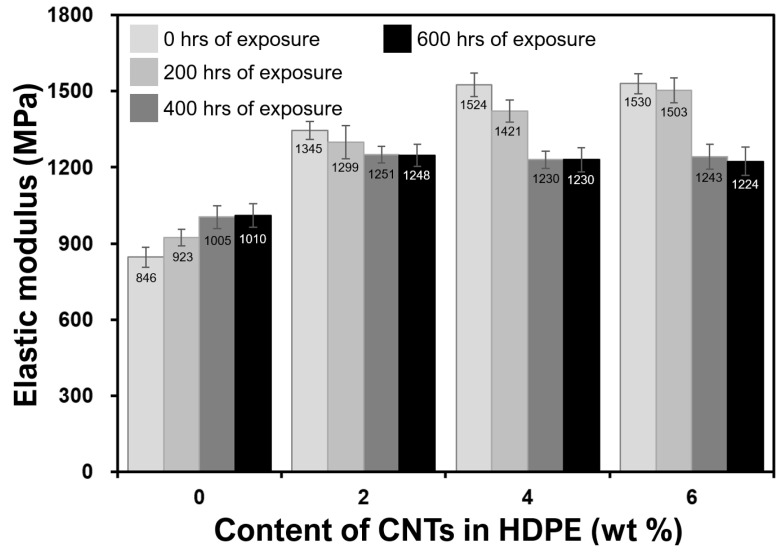
Effect of weathering on elastic moduli of monolithic and nanocomposite samples.

**Figure 4:**
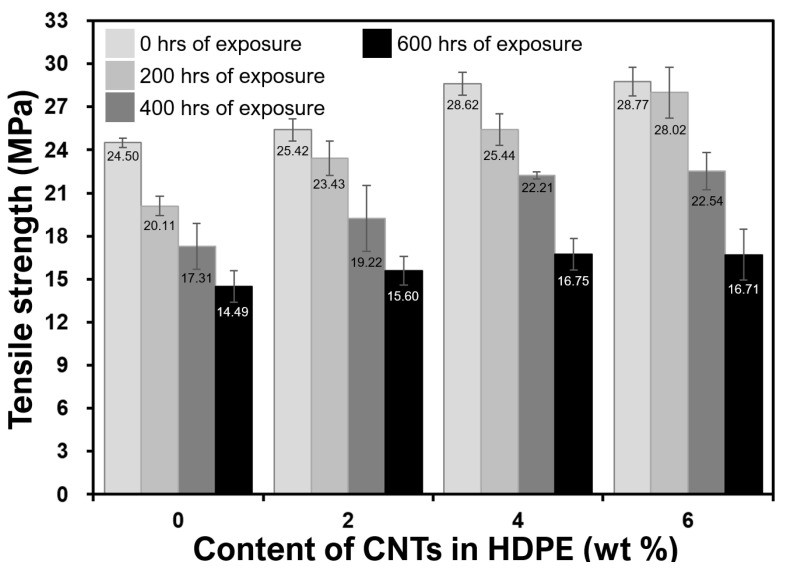
Effect of weathering on tensile strengths of monolithic and nanocomposite samples.

Mechanical properties from tensile testing of the neat HDPE and CNT filled nanocomposites are shown in Figure 3; Figure 4. The data presented are the averages of at least five samples. As compared to neat polymer (0 wt%), the elastic moduli (Figure 3) of all the unweathered nanocomposites improved (e.g., 81% for 6 wt% nanocomposites) because of the presence of CNTs, which stiffen the polymer matrix, as confirmed previously [9,10,23,25,29,30]. Similarly, as compared to neat polymer (0 wt%), the tensile strengths of all the unweathered nanocomposites improved (e.g., 17% for 6 wt% nanocomposites) because of the presence of CNTs. The enhancement in the tensile strength (Figure 4) can be attributed to better stress transfer that resists breakage, giving superior strength to the nanocomposites, as confirmed in number of previous studies as well [9,16,23,25,31].

From the analysis of the effect of weathering, it can be seen that the UV radiation caused embrittlement/stiffening for the neat polymer and reduction of moduli for the nanocomposites (Figure 3 and Figure 4). It is widely known that HDPE gains its mechanical strength from its long chains of polymers. The UV radiation (mimicking sunlight) attacks the polymer by breaking it into smaller chains, typically known as chain-scission [32,33,34]. The longer the exposure of UV radiation for a neat polymer, the lower the strength of the material (Figure 4). However, CNTs help to increase the strength and modulus and lower the weathering damage by an absorption mechanism. This has also been reported in other types of polymers, where CNT fillers stabilized epoxy and polyurethane matrices against UV-induced environmental degradation [35,36]. With CNTs, the UV absorption is taking place via vibrational transitions due to carbon–carbon bond stretching and other vibrationally active modes [37]. Breakdown of the polymer matrix by photoreaction or other weathering mechanisms can negatively affect mechanical properties of the HDPE by weakening interfacial interactions with CNTs (Figure 3 and Figure 4). This would also increase the potential for environmental release over the monolithic polymer.

From the analyses of Figure 3 and Figure 4, that there is a very minimal difference in improvements between 4 and 6 wt% compositions can be observed. CNTs are enhancing the elastic moduli and strengths of their respective HDPE nanocomposites. For 6 wt% nanocomposites, it can be noted that because of the presence of agglomerates, the modulus and strength are almost the same as those of 4 wt% nanocomposites. Furthermore, their ability to absorb UV radiations is almost the same or lower for 400–600 h of exposure and presented in Figure 3 and Figure 4. For instance, the tensile strength after 600 h of exposure was found to be 16.75 or 16.71 MPs for 4 wt% and 6 wt% CNT-filled nanocomposites respectively. The cause of such minimal or nearly no improvement in mechanical properties is due to the agglomeration, as evident in Figure 2d and reported in other publications [18,20,27]. Agglomerates (Figure 2d) reduce the surface area of CNTs interacting with the polymeric chains; hence, the mechanical properties were negatively affected (Figure 3 and Figure 4).

**Figure 5:**
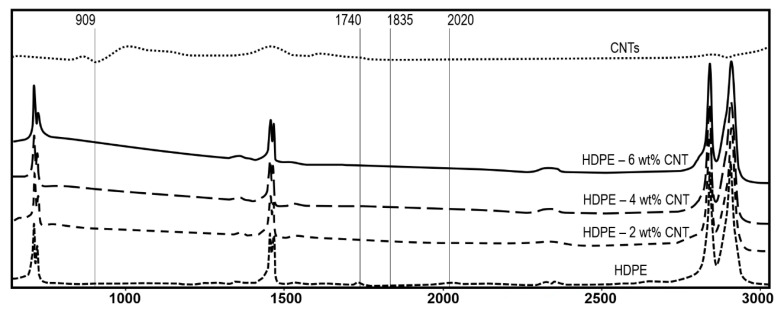
IR spectrum of HDPE, CNTs and nanocomposite films without exposure to UV radiation.

**Figure 6:**
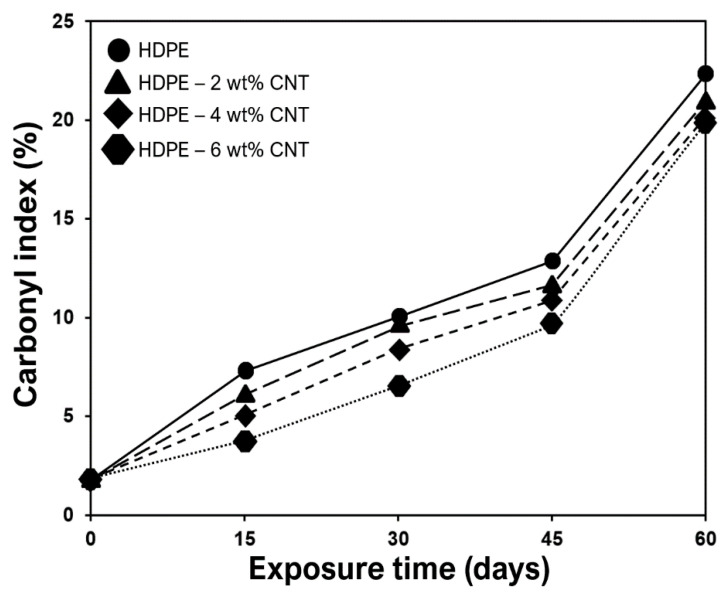
Carbonyl indices (*I*_CO_) of the monolithic and nanocomposite films exposed to UV-B radiation.

**Figure 7:**
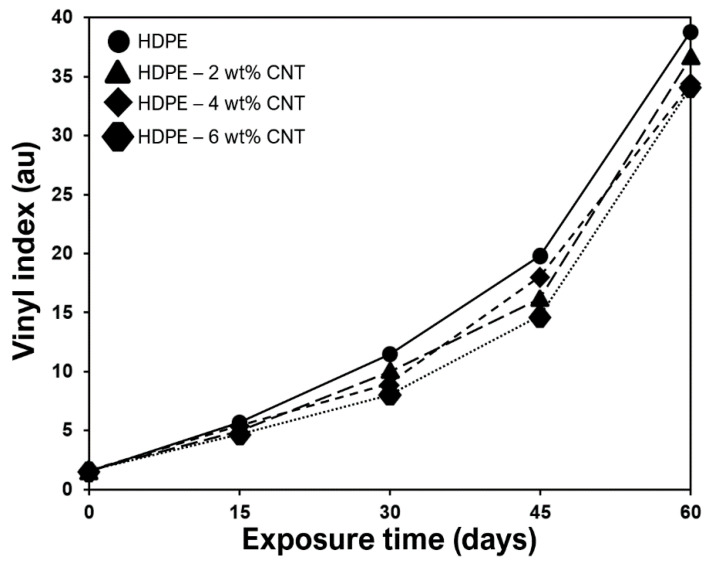
Vinyl indices (*I*_CO_) of the monolithic and nanocomposite films exposed to UV-B radiation.

The changes in structural properties of HDPE and CNT-filled HDPE nanocomposites exposed to UV-B radiation were studied (Figure 5, Figure 6 and Figure 7). The complete infrared spectra of HDPE, CNTs and nanocomposites without exposure to UV radiation are shown in Figure 5. After the UV light exposure, the carbonyl (*I*_CO_) and vinyl (*I*_V_) index were used to evaluate the photooxidation of these polymers and nanocomposites (Figure 6 and Figure 7). The carbonyl and vinyl groups, quantified by their respective absorption indices obtained from the IR spectroscopy (Figure 5), are considered the main photooxidation products for polyethylene [38,39,40]. The spectrum of HDPE presented typical bands of C–H group at 2923 and 2853 cm^−1^ (Figure 5) that were ascribed to asymmetric and symmetric stretching vibrations, respectively [41,42]. The presence of peaks between 1450–1490 cm^−1^, in both HDPE and nanocomposites, originate due to CH_2_ bending [43,44]. For raw CNTs, the peaks between 500–1000 and 1650 cm^−1^ are due to alkenyl C=C stretching [45,46], as presented in Figure 5. The bands between 2850 and 2922 cm^−1^ are associated with the symmetrical and asymmetrical stretching of CH_2_ groups [47].

*I*_CO_ indices were calculated using IR absorption band values of 1740 and 909 cm^−1^ respectively, as marked in Figure 6. The value 1740 cm^−1^ corresponds to the stretching vibration of the vinyl group (CH_2_=CH)*n*, whereas 909 cm^−1^ corresponds to the stretching vibration of the carbonyl group (C=O) [48,49]. Similarly, *I*_V_ indices were calculated using IR absorption band values of 1835 and 2020 cm^−1^ respectively, as elaborated in Figure 7. These absorbances values of 1835 and 2020 cm^−1^ are just reference values required to measure *I*_V_ indices. From the analysis of Figure 6 and Figure 7, it can be observed that CNT inclusion supresses the photodegradation of the respective HDPE nanocomposites due to absorption of UV radiation. Lower carbonyl and vinyl groups were observed for nanocomposite with higher CNT content (Figure 6 and Figure 7). *I*_CO_ indicates a higher level of polymer backbone scission [50,51]. *I*_V_ of HDPE increases with almost linear tendency due to the embrittlement process and breaking of bonds in the tertiary carbons of the branches of the polymer backbone [50,51]. It was also observed that after 60 days of cyclic exposure, the amounts of carbonyl and vinyl groups in 4 wt% and 6 wt% nanocomposites were almost the same.

**Figure 8:**
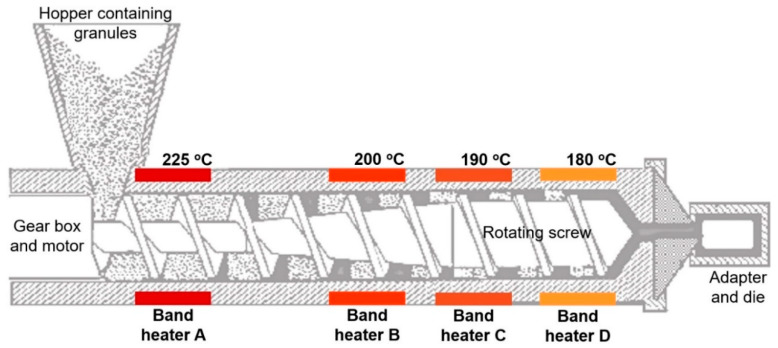
Schematic of extruder with heating temperatures used in this work.

**Figure 9:**
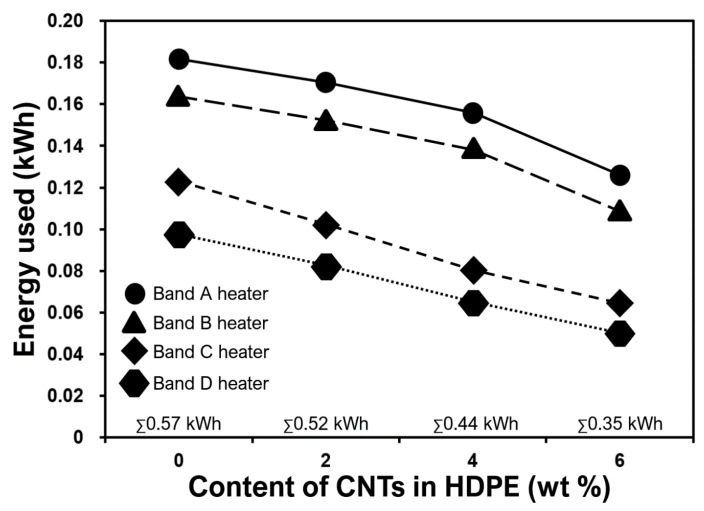
Energy used by respective band heaters for a complete 10 min extrusion cycle. The total energy, the sum (∑) of energy data points for every concentration, is on the horizontal axis line.

For successful adoption of these novel materials, energy consumed (by manufacturer just before the end-user) is a key business critical variable for bulk manufacturing of sheathing layer in an umbilical cable (Figure 1). Energy consumed by the band heaters (Figure 8) during the extrusion of CNT filled nanocomposites was compared with neat HDPE by calculating the electricity used for 10 min extrusion cycle. Figure 9 shows the energy used by respective band heater, as positioned in Figure 8 with the respective maintained target temperatures. The total energy, the sum (∑) of energy data points for every concentration, is on the horizontal axis line (Figure 9). It can be seen that, in comparison to monolithic HDPE, 4 wt% and 6 wt% nanocomposites used 23% and 39% less energy respectively. This is because of the high thermal conductivity of CNT-filled HDPE nanocomposites widely reported by other research groups [51,52,53,54,55,56]. Owing to the higher thermal conductivities of these nanocomposites, less energy was consumed by the band heater locally to achieve programmed target temperatures (Figure 8). Hence, it can be concluded that lower energy is required to mass-extrude CNT filled thermoplastic nanocomposites, making them energy friendly for offshore sheathing layer in an umbilical cable.

## 3. Materials and Methods

A commercial master batch grade of nanocomposite granules, PLASTICYLTM HDPE1501, supplied by Nanocyl SA, Sambreville, Belgium, was used in this study [57]. PLASTICYL HDPE1501 is a masterbatch based on HDPE loaded with 15 wt% of MWCNTs. According to the supplier, the masterbatch contains CNTs (diameter ranging between 30 and 80 nm, 1.5 mm average length, purity 90% by TGA, NC700 series) synthesised by catalytic carbon vapour deposition method. HDPE commercial grade, BorPure MB7541, supplied by Borealis Plastomers B.V., Sittard-Geleen, Netherlands, was used to prepare nanocomposite samples of the required compositions.

The masterbatch containing 15 wt% CNTs was carefully compounded with the HDPE matrix in a twin-screw extruder to produce nanocomposite concentrations of 2 wt%, 4 wt% and 6 wt%. A twin-screw extruder was used to ensure good dispersion and chemistry integrated within the masterbatch by the commercial supplier. The extruded strands were pelletized into smaller granules followed by vacuum drying at 70 °C for 24 h. The next step was the actual extrusion of HDPE and the nanocomposite materials. Melt intercalation was performed with Brabender PL-19 single screw extruder (Brabender Measuring Extruder 19/25D, supplied by Brabender GmbH and Co. KG, Duisburg, Germany) at screw speed of 95 RPM and temperatures between 180 and 225 °C. A rectangular shaped die (80 × 2 mm), with a unique funnel design was used. The extrudate was received as rectangular strip samples from which standard dumbbell-shaped samples were punched out. Figure 8 shows a schematic of the extruder elaborating the locations and temperatures of the band heaters used in this research. During the extrusion process, the extruder variables (screw speed and temperature) were systematically optimised to ensure consistency of the extrudate quality. For consistency purposes, once the target temperatures were achieved, a 10 min cycle of extrusion (yielding around 3 m of extrudate) was used to calculate the energy consumed in this comparative study. After every batch, the extruder barrel was thoroughly cleaned using HDPE granules, according to the standard operating procedures provided by the manufacturer. The energy consumed, for comparative analysis, was measured using Equation (1). Kilowatt-hour is a composite unit of energy equal to one kilowatt of power used for one hour.

Energy consumed = Watts × time (hrs)/1000(1)

The dumbbell-shaped monolithic and nanocomposites samples were subjected to 200–600 h of accelerated weathering test using Weice Xenon test chamber. The accelerated weathering test was carried out in compliance with the standard ASTM G155–13 and the variables were carefully selected to imitate the real offshore conditions. UV radiation and wavelength were set to 0.60 W/m^2^ and 342 nm respectively. The details of the 200 h weathering cycle used for the test are shown below. Here, 2 and 3 cycles of 200 h were used for completing 400 and 600 h of weathering respectively:

40 min light, 65% relative humidity (RH), 60 °C;
40 min light and sea water spray on specimen, 95% RH, 40 °C;
60 min light, 65% RH, at 60 °C;
60 min dark and sea water spray on specimen, 95% RH, 40 °C.

The mechanical characterisations of the monolithic and nanocomposite samples were performed at 21–23 °C with humidity levels ranging between 52% and 60%. The tensile properties were measured according to ISO 527-2 (5A) using an Instron testing machine (model 6800 using advanced video extensometer, AVE 2).

The morphological analyses for the monolithic and nanocomposite samples were carried out using a transmission electron microscope (Jeol 2010, Jeol Ltd., Tokyo, Japan) with an accelerating voltage of 100 kV. The samples were thinned to around 70–100 nm in thickness using Leica Artos3D ultra-microtome technology having a 4 mm high precision diamond knife.

For measuring carbonyl (*I*_CO_) and vinyl (*I*_V_) indexes (photodegradation analyses), monolithic and nanocomposite films (having thickness of 50 microns) were prepared using ultra-microtome technology. Samples were mounted around 5 cm away from the lamp and analysed at regular time intervals of 0, 15, 30, 45, and 60 days. UV radiation and wavelength were set to 3 W/m^2^ and 342 nm respectively. The samples were then characterised by IR spectroscopy with Attenuated Total Reflectance (ATR), using a Nicolet iS10 FTIR spectrometer (supplied by ThermoFisher Scientific, Paisley, UK) having a Ge mirror and a resolution of 2 cm^−1^ in the range of 700–2500 cm^−1^. IR absorption band values (AXXX) corresponding to respective stretching/vibration of key molecular groups were obtained from the IR spectroscopy. Carbonyl (*I*_CO_) and vinyl (*I*_V_) indices were calculated using Equations (2) and (3) respectively [48,49].

*I*_CO_ = (A1740 − A1835)/(T × 0.008) (2)

*I*_V_ = A909/A2020 (3)

where T is the thickness of the sample in mm.

## 4. Conclusions

CNT-filled HDPE nanocomposites with 4 wt% and 6 wt% loadings are the most optimal concentrations, as they provide a good balance between mechanical properties and resilience of mechanical properties against UV exposure for the offshore umbilical sheathing layer (Figure 1). Good dispersion was achieved for nanocomposites up to 4 wt% CNT loading. The mechanical properties (elastic modulus and tensile strength) of all the unweathered nanocomposites improved because of the presence of fibrous CNTs aligned with the direction of extrusion. For weathered nanocomposite samples, CNTs aided in supressing the matrix damage, which resulted in superior mechanical properties as compared to neat HDPE. Little or no difference in mechanical properties (before and after weathering) was observed between 4 wt% and 6 wt% filled nanocomposites. CNTs also suppressed the photodegrading in HDPE nanocomposites by creating lower photodegradation products (carbonyl and vinyl groups). In comparison to neat HDPE, lower energy is required to mass-extrude CNT filled thermoplastic nanocomposites, making them attractive (energy friendly) for offshore sheathing applications.
